# A Time Study Analysis of Fluoride Varnish Application in Pediatric Well Visits to Address Health Disparities among Children

**DOI:** 10.22105/jarie.2024.436316.1595

**Published:** 2024-06

**Authors:** Robert S. Keyser, Emily Rodriguez-Jacobo, Christina Scherrer

**Affiliations:** 1 Department of Industrial and Systems Engineering, Southern Polytechnic College of Engineering and Engineering Technology, Kennesaw State University, Marietta, Georgia, USA;

**Keywords:** Fluoride varnish, Dental caries, Primary care, Time study, Standard time

## Abstract

Dental decay is the most common chronic disease in children. Fluoride varnish (FV) is a preventive oral health service with proven effectiveness at reducing dental caries in dental and primary care settings. The objective of this study was to determine how long it takes to apply FV treatments during primary care well visits to address one of the most common barriers as reported by pediatricians – lack of time. FV treatment videos were collected at six clinics in Georgia with rigorous time studies conducted on each video to determine the Standard Time for the FV treatment process as well as the FV Application Component of the process and reasons for delays. Median Standard Times varied by clinic, ranging from 67.7 seconds to 166.9 seconds with an overall median of 109.7 seconds. This results in per FV application labor costs of approximately $2.38 for pediatricians, $1.16 for registered nurses, and $0.53 for medical assistants. Findings from this study support the inclusion of FV applications as a common practice during primary care well visits.

## 1| Introduction

The objective of this study is to address one of the most common barriers to offering FV treatments during a well visit as reported by pediatricians – lack of time – by: (1) obtaining an accurate estimate of the time required to complete FV application during a well visit, (2) determining the value-added (VA) ratio of the actual FV application time as a proportion of total FV process time, and (3) examining reasons why some FV applications may take significantly more time than others. In the context of a busy pediatric well visit, the time of the providers and support staff is a significant resource that must be optimized. Various sources suggest that FV application “took no more than 2–3 minutes” [1], [2], [3], [4], but there is a need for more exact estimates of the time involved with all aspects of FV application. Our utilization of time study in fluoride varnish applications has not previously been used to this degree in a medical setting and thus expands the body of knowledge related to time study application.

## 2| Literature Review

Dental caries, also known as dental decay, is the most common chronic disease in children [5], affecting 33.3% of children ages 3 to 5 and over 54.4% among those 6 to 9 years old [6]. The U.S. Surgeon General called dental caries a “silent epidemic” [7]. Significant adverse outcomes are associated with untreated dental caries leading to diminished quality of life [8] and overall well-being of the child [9] including pain, tooth sensitivity, tooth abscesses, broken teeth [10], tooth loss, speech impediment, compromised self-esteem [11], dysfunction, poor appearance, low concentration on daily life achievements [12], school absenteeism and poor performance [13], and, in extreme cases, death [14].

Access to dental care is a major public issue, especially for racial minorities and children living in poverty [15], [11]. Children 5 to 19 years of age from poor and racial or ethnic nonminority families have higher rates of untreated dental caries than their counterparts from non-poor and nonminority families [16]. Inadequate access to dental care among racial or ethnic minority populations may be attributed to lack of health insurance coverage [17] due to low socioeconomic status [18], geographic maldistribution of dentists and pediatricians in communities where oral health services are needed the most [19], and severe shortages of minority dentists who are more likely to accept Medicaid to serve these communities [20]. This lack of access to preventive oral health care for young children leads to more costly restorative care which places a significant financial burden on families.

Fluoride varnish (FV) is a preventive oral health service with proven effectiveness at reducing dental caries in dental as well as primary care settings [21], [22]. Evidence supports between two to four FV applications annually as an intervention that can reduce dental caries in primary teeth among young children [23], [21]. The application of FV, which is a protective coating painted on teeth to prevent tooth decay, requires no special equipment and is a very simple procedure. Studies show that topical fluorides [24] and semi-annual FV applications are effective in reducing caries increments of first permanent molars [25]. The American Academy of Pediatric Dentistry [26] and the American Dental Association [27] recommend FV use for children considered “at risk”, which includes all children with low access to dental care.

Because dental caries is such a common disease in children, and since young children are more likely to visit medical offices than dental offices [15], it is essential that pediatricians include oral health in their child well visits [28]. The U.S. Preventive Services Task Force [29] recommends non-dental providers apply FV to all children under 5 years of age and as early as 6 months of age [28], during well visits to prevent early childhood caries. Since 2017, all state’ Medicaid/CHIP programs reimburse FV applications in the primary care setting, yet only a small proportion of primary care providers (PCPs) apply pediatric FV [29]. However, despite two studies by [30], [31] citing evidence that FV applications during medical visits improve oral health, fewer than 1 in 10 child well visits include FV treatment [2], [15], [29], [32].

FV treatment barriers reported by pediatricians include not enough training, inadequate time during well visits, hesitancy on where to find supplies [33], [34], insufficient Medicaid reimbursements [35], cost [33], and profitability for the office [36]. Prior estimates of time have lacked rigor [1], [2], [3], [4] which can contribute to these barriers of implementation.

Time study is a quantitative study of a task while it is performed by a trained time study analyst. It is an appropriate measurement for manual work that can be physically observed by the analyst as well as for repetitive tasks that can usually be done in 15 minutes or less. It is the most versatile and most widely used work measurement technique in the industrial engineering literature [37], [38]. The following paragraphs indicate how time-motion study has been applied in healthcare applications, split into three categories: (1) How time-motion study is used to assess methods changes; (2) How time-motion study is used to compare the effect of technology changes in processes vs. a traditional way; and (3) How people spend their time in various occupations.

Young et al. [39] studied the administrative workload associated with electronic health records and measured how much time physicians and interns spend in various parts of their jobs. Chaiyachati et al. [40] assessed general medicine inpatient time allocation among first-year internal medicine residents. Whereas Deer et al. [41] performed a motion study to assess the effects of a *mild*^®^ procedure as a first-line therapy in combination with nonsurgical conventional medical management (CMM) vs. CMM alone on patients with lumbar spinal stenosis suffering from neurogenic claudication secondary to hypertrophic ligamentum flavum, Bari et al. [42] used motion study to investigate differences in gait patterns among individuals with different walking speeds. Singhal et al. [43] applied time-motion study to examine when and how heart failure processes occur to identify areas for improved efficiency with a primary finding of most patient time was spent in travel and waiting between activities on the day of their heart failure appointment. Lin et al. [44] applied time-motion study to compare subcutaneous vs. intravenous trastuzumab mono-therapy treatment options for early-stage breast cancer patients. Bawden et al. [45] studied the use of time-consuming interventions, such as procedural sedation and analgesia, on patients in a Canadian emergency department. Whereas Dolan et al. [46] applied time-motion study to investigate efficiency changes in immunization clinic workflow design, moving from the existing dual-data entry workflow to a future paperless workflow, de Hond et al. [47] performed time-motion study to explore differences in actual administration time of medication to patients vs. the documented time in the electronic health record (EHR) and Meguerditchian et al. [48] used time-motion study to evaluate the number, different types healthcare professionals involved, and the time it took to complete tasks related to medication reconciliation in the admission and discharge processes in three hospital units. Smith & Gale [49] employed time-motion study to explore the complexities of discharge, the length of time taken, and evaluation of time wasted. Webster et al. [50] performed time-motion studies to estimate the time and costs associated with an unoccupied bed in a hospital facility.

Mohammed et al. [51] used time-motion study to determine time savings in actual wound assessments utilizing Artificial Intelligence (AI) vs. manual methods. Innovative software has been employed in interventions to make faster triage evaluations and recommended procedures. Examples include Kinovea software to assess weaknesses and shortcomings in athletes’ movements [52] and ARGOS^®^ to demonstrate efficiencies in cataract evaluation and surgery [53]. Time-motion studies have been used to assess the radio communication between inbound ambulances and emergency department (ED) personnel to determine how often radio reports result in actions taken by ED personnel to prepare for patient arrival [54]. The use of EHRs has been time-studied to evaluate the time spent by patients on different tasks during their visit to primary healthcare centers [55].

Time-motion studies have been used to track movements and tasks of nurses [56; 57], how physicians spend their time providing care in an ambulatory practice [58] and emergency departments [59; 60], how pharmacists spend their time during the discharge medicine handover process generating medicine lists [61], and how multidisciplinary teams use their clinical time [62]. Additionally, time-motion studies have explored workload requirements of multipurpose healthcare workers [63; 64], and accident and emergency consultants [65], and community health workers [66; 67].

However, the above studies were intended to determine the fraction of time spent in various activities and did not consider any allowances for fatigue or identify any foreign elements. To the best of our knowledge, time study has not been used to estimate the amount of time that a medical procedure takes for the purpose of decision-making within a practice. The high value and limited resource of provider time necessitates this work. Whereas the application of time-motion study is prevalent in the healthcare industry, in general, and medical research, specifically, the literature also did not reveal any publications regarding the use of time-motion studies in pediatric practices.

## 3| Methods

### 3.1| Recruitment and data collection

The principal investigator (PI) coordinated recruiting with primary care practices and clinics (referred to hereafter as ‘clinics’) currently applying FV throughout the state of Georgia, ultimately forming research partnerships with six clinics to collect time study data. Recruitment was intentionally done to obtain a mix of urban, suburban, and rural clinics and representation from a variety of practice sizes and experience levels. As a small fraction of clinics in the state are currently applying FV, recruitment was done through a combination of cold calls from the PI and connections from advisory board members participating in the research project. Although specific clinic names are not included to protect their anonymity, the coded participant clinics in this study are: A, B, C, D, E, and F. Four of the six clinics (A, B, C, and E) are in the greater metro-Atlanta area. Clinic F is in a rural area well north of Atlanta where healthcare access is not as prevalent as in urban areas and Clinic D is a Federally Qualified Health Center in the southern part of the state. Clinics A, B, and C have consistently been applying FV for multiple years, and each of the providers at those practices who were part of the data are also experienced in FV application. Small practices were defined as those with two or fewer full-time equivalent doctors, physician assistants and certified nurse practitioners, and large defined as those with more than ten. A clinic which had been consistently applying FV for at least one year during pediatric well-child visits was classified as “established”. Clinic D has recently restarted after pausing during the COVID-19 pandemic, Clinic E had recently begun applying FV for the first time, and Clinic F applies FV somewhat inconsistently and during separate well visits. This study was approved by the university’s Institutional Review Board. Each provider participating in the research was required to sign an Informed Consent Form. Caregivers of children participating in the study received an Informed Consent Cover Letter, which did not require a signature, as no personally identifiable information about the child was collected as part of the study.

Videos were collected using either an iPad or video camera device, dedicated to the research project. Each video was coded by clinic (i.e., A1 for the first video collected at clinic A) and was designed to capture all the activities involved in administering the FV treatment by the provider. Student researchers were invited into the room to obtain consent from caregivers. Video recording began when the provider started to get materials ready and concluded once the FV was completely applied and the provider moved to another task. Once the video process was completed, the researcher exited the exam room so that the provider could complete the child well visit. The researcher uploaded the videos to the university-secured OneDrive folder and then promptly deleted the video from the recording device. Video collections from the six clinics took 12 months to complete.

### 3.2| Time studies procedure

Student researchers performed time studies on each video with different students performing first and second reviews (i.e., A1_1 and A1_2). Students received time study training via an undergraduate Work Measurement Study course in their Industrial Engineering major at the university as well as under the mentorship of one of the co-PIs throughout this study. The first video was reviewed by the team together to ensure that everyone was following the same process and students were able to ask any questions and clear up any uncertainties they may have. The FV process in each coded video was divided into separate and distinct work elements that were sequentially arranged on a time study worksheet. A typical time study included the following five work elements: don gloves, open FV packet, FV method (i.e., dab FV on wrist and then onto applicator), apply FV, and wipe mouth. Be designating consistent starting and stopping points for each work element, observed times for each work element were obtained and recorded. Student researchers also accounted for anomalies, considered “foreign elements”. Foreign elements are any unusual, non-routine, activities that accounted for a delay in the process. Performance ratings were assigned for each work element, based on the time study analyst’s best judgment from familiarity with what constitutes a “normal pace”. For example, a work element that was performed at a “normal pace” resulted in an assigned performance rating of 100%. If a work element was performed at a “slower than normal pace,” the performance rating was less than 100%. Conversely, if a work element was performed at a “faster than normal pace,” the performance rating was greater than 100%. Performance ratings were assigned by the time study analyst in multiples of 5%.

The base time for each work element was determined by multiplying performance ratings by the average time for each work element. The normal time for each work element was determined by multiplying the base time for each work element by the frequency of each work element and foreign element, in relation to the overall unit of measurement – one FV treatment. The Total Normal Time consists of the sum of all work element and foreign element normal times. An allowance factor for personal time, fatigue, and delay (Apfd) was assigned by the time study analyst based on the analyst’s observations of the video. The Apfd was multiplied by the Total Normal Time to determine the allowable time for personal time, fatigue, and delay activities. The Total Normal Time plus the Apfd time yielded the Standard Time for the FV treatment process. The Standard Time represents the time it should take an average trained individual, following standard procedures, and working at a normal pace, to perform the FV treatment, with a built-in allowance factor for personal time, fatigue, and delays. It is the best representation of the time that would need to be allocated to FV application in a well visit. Hence, this “Standard Time” is the primary measure studied in the analysis below. In addition, separate analyses were performed for the “FV Application Component” of the process in isolation and as a value-added ratio of the Standard Time.

In cases where the two time study analysts obtained results that differed by more than 15 seconds a third time study was performed. Results of the three time studies were then evaluated and the two time studies that aligned best in terms of similar work elements and foreign elements (i.e., delays) were retained, with the third time study discarded. If the third study were to also differ from the others, the Co-I who has significant experience in time study would have resolved the difference, but that issue did not arise.

### 3.3| Statistical analysis

To determine the desired sample size, the sample standard deviation of Standard Times was averaged for the first 10 videos collected, which were from two different clinics. An average of 16.99 seconds was obtained. With an objective margin of error of 15 seconds at confidence level 95%, a total sample size of 5 videos per clinic was determined necessary. However, the team hypothesized that standard deviations may be higher at other clinics and wanted to be able to detect differences among various factors, so the team attempted to collect 10–20 videos from each clinic. At the conclusion of our 12-month video collection period, a total of *n* = 94 videos was obtained (A = 14, B = 20, C = 20, D = 14, E = 19, and F = 7). The team was limited to two visits to clinic F, which prevented collecting 10 videos there. It was determined to keep clinic F in the analysis due to its rural location, relatively small standard deviation in times, and sample size larger than 5.

The software used for our statistical analyses includes SPSS v29, Minitab v21.3, and Excel. Kendall’s’ *τ* test was performed to validate interrater reliability between Analyst 1 and Analyst 2 for the retained analyses in this study. Additionally, we observed and recorded the actual time required to apply FV as well as the various reasons for delays during the entire treatment process. ANOVA was used to determine the impact of main effects and their interaction terms on the response variable, Standard Time, and then separately on FV Application Component. Seven fixed factors include X1 = Clinic (A, B, C, D, E, F), X2 = FV Method (FV dab on wrist to applicator, applied by applicator directly from packaging, or applied with gloved index finger directly from packaging), X3 = Child’s head positioning (reclined on caregiver with head in provider’s lap, held in upright position by caregiver (lap or standing), sitting upright unassisted (exam table or chair), or reclined on exam table), X4 = Child’s cooperation level (easy, moderately difficult, extremely difficult), X5 = Apfd, or allowance for personal time, fatigue, and delays (5%, 10%, 15%, 20%), X6 = Gender (male, female), and X7 = Well visit age (<12 months, 12 month, 15 month, 18 month, 2 year, 3 year, 4 year or older). Potential factors to study were determined by the researchers and the project’s advisory board, consisting of pediatricians, public health professionals, dentists, and an insurance representative. The model includes patients as a random factor. All main effects and relevant two-factor interactions were analyzed at the *α* = 0.05 significance level and Tukey’s post hoc test was completed, where appropriate.

We also provide estimates of FV delivery costs from the provider’s perspective. Material costs, estimated from interviews with office managers and searching internet medical supplies stores’ prices total approximately $1.25 for the FV application kit, one pair of gloves, and a gauze pad to wipe the mouth. Median hourly labor costs of $78.12 for pediatricians, $38.19 for registered nurses, and $17.54 for medical assistants were obtained from 2022 data from the U.S. Bureau of Labor Statistics [68]. We did not include indirect costs, due to their variation by independent practice characteristics. The reimbursement rate from Medicaid/CHIP in Georgia for fluoride varnish application is $17.59 [69]. Office managers shared that private insurance typically reimburses at a higher rate, so that serves as a conservative estimate for reimbursement rate.

## 4| Findings

Of the 94 patients, approximately half were male (48%), though there was variability among clinics. (See Table 1). The relationship between clinic and gender was not found to be significant (p-value = 0.11). At least 9 videos were collected from each well visit age, and the 12-month visit was most common with 26 videos (28%). Notably, clinic B intentionally only applies FV at 12-month, 18-month, and 2-year visits while the other clinics apply FV at any well visit where the child has teeth, has not received FV in the last 6 months, and is not yet connected to a dental home. Well visit age was found to be correlated with positioning (p-value = 0.04), but with no other factors.

Table 2 summarizes the methods used by each clinic, as well as the clinic characteristics. Approximately three quarters of applications (76%) involved FV applied with the applicator directly from the packaging. Both providers at one practice (A) put a large amount of varnish near their wrist on the thumb side of the glove and applied with the applicator from there. One provider at another practice (B) used that method only for her younger patients (n = 2). One provider (practice D) used her finger to apply the FV for all patients (n = 7). The method of FV application was found to be significantly correlated with clinic (p-value < 0.001).

As noted above, patient positioning had a high correlation with age. Approximately 90% of patients at their 12-month visit and younger were either reclined on their parent’s lap with head in provider’s lap or laying down on the exam table – split relatively equally between the two methods. The rest were held in their parents’ arms. The preference between these two positions for the youngest patients appeared to be somewhat clinic-dependent, but the relationship between positioning and clinic in general was not found to be significant (p-value = 0.576). Starting at the 2-year visit, some children sat independently, and by 4 years, 79% of children were sitting unassisted, with the remainder laying down on the exam table.

We analyzed both the Standard Time to complete FV application, including opening the packaging, positioning the child and delays related to those elements and the FV Application Component, which was the time to apply the FV and delays that occurred in that part of the process. The median Standard Time across all clinics was 110 seconds (see Table 3) and the mean was 130.1 seconds (95% CI = 116.4, 143.9). The median FV Application Component was 32.4 seconds with mean 46.5 seconds (95% CI = 39.0, 54.0). We also measured the value-added ratio of actual FV Application Component as a proportion of the standard time and found the median to be 29.6% and mean 35.7%. Kendall’s *τ* results of 0.940 (p-value <0.001) for Standard Time and 0.801 for FV Application Component (p-value <0.001) indicate strong interrater reliability (i.e., correlation) in measurement readings between Analyst 1 and Analyst 2.

A median standard time of 109.7 seconds represents median labor cost in Georgia of $2.38 for pediatricians, $1.16 for registered nurses, and $0.53 for medical assistants [68]. Adding the materials cost of $1.25, this results in a range of direct costs from $1.78 to $3.63 per application, depending on the human resource used for the FV process. At five of the participating practices, the registered nurse, nurse practitioner, or pediatrician applied the FV and at one location FV application was completed by the medical assistant.

**Fig. 1.**
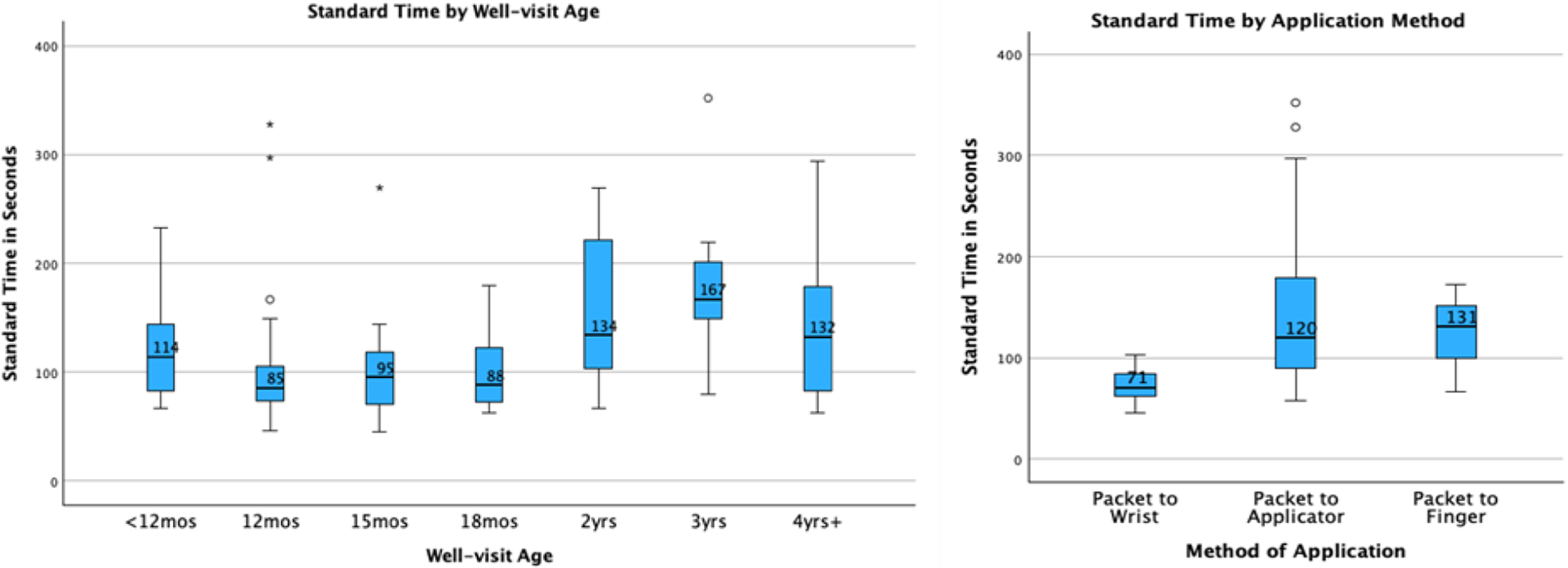
Box plot of Standard Times for each well visit age and application method.

An ANOVA analysis was performed on Standard Time as a factor of nominal variables clinic, gender, FV method, and positioning and ordinal variables well visit age, cooperation level, and Apfd. The only variables that were statistically significant were clinic (p-value <0.001), FV method (p-value <0.001), and age (p-value = 0.012). No statistical significance was found from the second order interactions. A Posthoc Tukey test found that the Standard Times for clinics A, B, and C were statistically lower than those of clinics D, E, and F. Those three clinics are the established practices. Their mean Standard Time was 100.9 seconds (95% CI = 89.3, 112.4) and their median 89.5 seconds. The Tukey test for age found Standard Time to be shorter for the four well visit categories of 18-month visits and younger than it was for 3 year visits, but other differences (including 2 year and 4+ year visits) were inconclusive. Finally, the dab on wrist method was found to be faster than the other two methods of FV application in this data. Figure 1 illustrates some of these differences graphically through box plots.

**Fig. 2.**
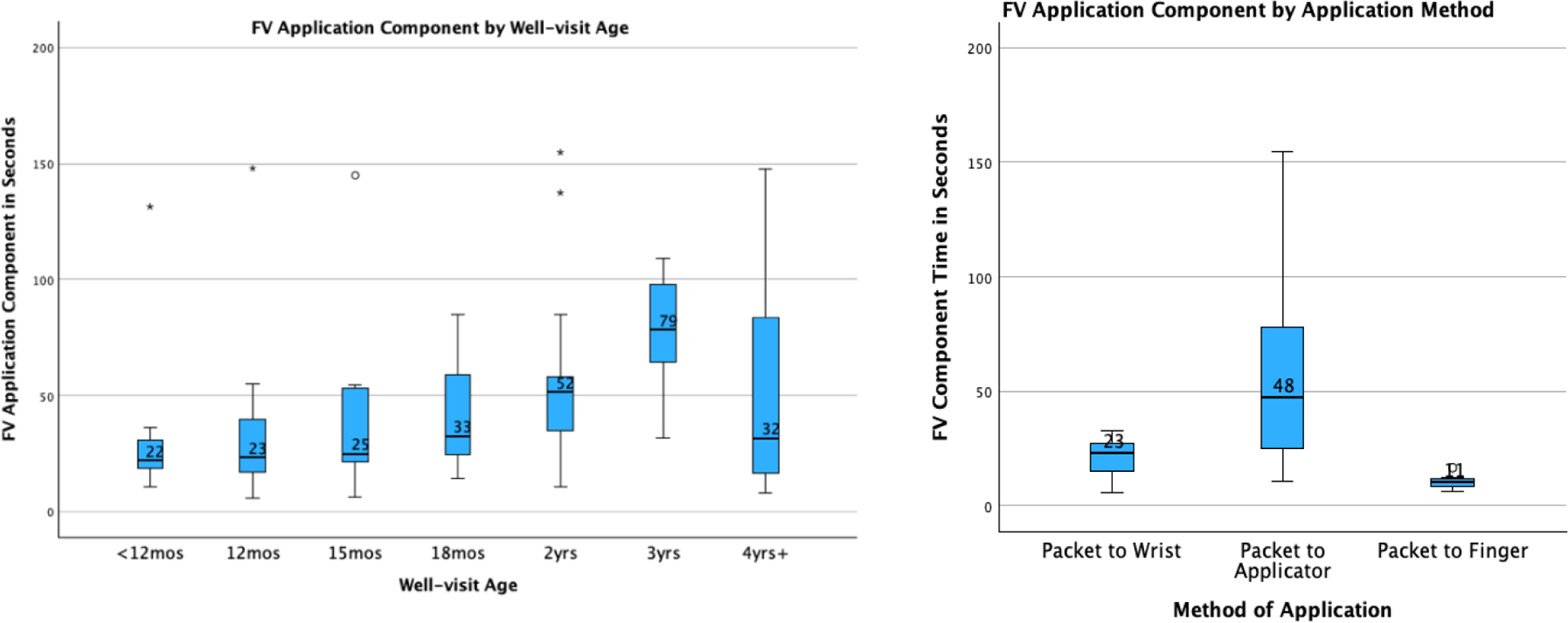
Box plot of FV Application Component Times for each well visit age and application method.

When analyzing the FV Application Component time, the same three factors were found significant: clinic (p-value <0.001), FV method (p-value <0.001), and age (p-value <0.002). In this case, clinics A and B had faster times than C, D, E and other differences were not statistically significant. The findings for age groups were the same as Standard Time, and the FV method from the packet to the applicator was found to be slower than the other two methods. A box plot of Standard Times for each well visit age and application method is found in Figure 2. Medians for each age are displayed on the graph.

**Fig. 3.**
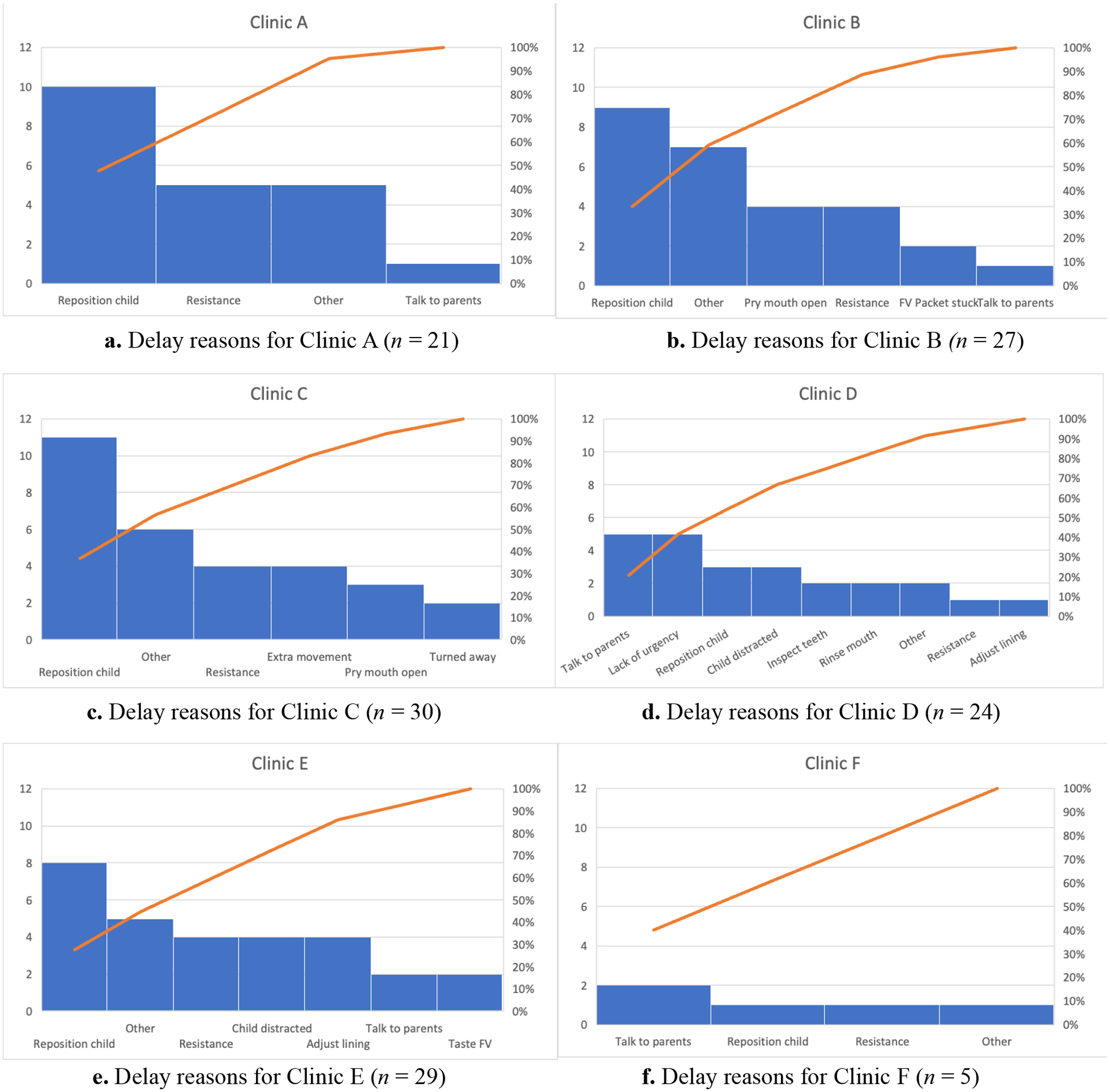
Summary of primary delay reasons that contribute to variation in Standard Times by clinic.

In any procedure, foreign elements, such as delays, are to be expected. An analysis of the common delay reasons by clinic can be found in Figure 3. A total of 136 delays were noted by the time study analysts, or an average of 1.45 per patient. The most common cause of delay was needing to reposition the child, followed by child resistance, and additional conversations with parents.

## 5| Research Limitations

Further work is warranted in addressing the limitations of this study. Specifically, the study was limited in being able to collect data from six sites currently applying FV in the state of GA. An increase in sample size of the number of time studies across different clinics in both urban and rural settings and in different states would allow for more generalized standard FV application times in primary care clinics as well as more nuanced study of interactions between age, clinic, and other factors. Our inclusion of one clinic with a smaller number of videos at one of our rural sites is a limitation of the results in that area. While the factors studied were approved by an advisory board of experts, it is still possible that additional important aspects impacting the time for application were not included in this study. An interesting follow-on study would be to collect data from the inexperienced practices in the future to verify that their time of application has reduced to the level we found in the experienced practices here. Additional studies could also examine ways to reduce the current average found of approximately 2 minutes FV Standard Time by establishing a standard procedure for administering this treatment. Further work can also investigate inadequate access to preventive oral health services for underserved populations, children living in poverty, and special needs children since this generally leads to higher risks of dental caries among children.

## 6| Practical Implications

Our novel research applies time studies to FV treatments in primary care settings. This is the first study to rigorously study that time, versus relying on anecdotal evidence. These results are important, since lack of time is a primary reason given by pediatricians for not applying FV. For example, “inadequate time” was selected as either a “moderate” or “significant” barrier to providing oral health services for 28.8% of providers in a 2012 American Academy of Pediatrics survey [33] and was a top concern in focus groups among providers and staff who had recently received FV application training [70]. The National Network for Oral Health Access [71] also noted time and cost as challenges in their implementation of FV application programs. Our findings should provide confidence to primary care providers and practice managers that FV application can fit into the well visit without extensive time required. For example, if a clinic schedules 30 minutes for well visits, FV application would take, on average, approximately 7% of the well visit time. In addition, the direct cost from labor and materials ranged from $1.78 to $3.63 per application, depending on the labor resource used to apply FV. Different states have different practice act requirements, and in some environments the pediatrician may prefer to apply the FV while doing an oral health risk assessment, whereas in other practices it may be more practical for medical assistants to apply FV along with their routine vitals or vaccine schedules. Some practitioners will include oral health counseling with the FV application, and depending on the state, counseling would have an additional reimbursement. Regardless of how the practice prefers to operate, the direct costs are significantly lower than a conservative estimate of reimbursement rates of $17.59 per application for Medicaid patients (higher for private patients and lower for uninsured).

Importantly, our findings validate anecdotal estimates in the literature, i.e., FV treatments took no more than a few minutes, on average, regardless of clinic or location (urban vs. rural). For example, Gnaedinger [1] found through surveys of providers following a quality improvement project of a FV application program in a Vermont pediatric practice that the FV application process seldom took more than 3 minutes. Similarly, Sudanthar et al. [3] found resistance raised about the time commitment for the implementation of FV for all children during another quality improvement project, but that the actual application time was approximately 60 seconds. Finally, for a small sample of 9 children in a South Carolina Rural Health Center, a mean time of 1 minute 57 seconds was reported for the application of FV [4]. Our results provide a more accurate estimate of median FV application process time (Standard Time) of 110 seconds and FV Application Component time of 32 seconds.

Our analysis determined that clinics A, B, and C were significantly faster than the others. The weighted mean Standard Time for those three clinics was only 100.9 seconds, versus an overall mean of 130.1 seconds. This is not a surprise, since clinics A, B, and C have been applying FV for a long time and are very consistent at including it during well visits. In contrast, clinic D has recently resumed FV application following a halt during COVID, clinic E began applying FV only a month prior to the start of data collection, and clinic F follows a non-traditional approach to FV application during each well visit. Inexperienced providers, in general, may take a longer time to explain the process to parents and have less confidence in completing the process. They may also feel less urgency if they are carefully going through a new process. In addition, seasoned FV providers make take less time to gain child compliance than less seasoned FV providers and have fewer difficulties with repositioning the child.

Both between and within clinics, we observed variability in the FV method and positioning. The standard method of FV application is from the applicator included in the packet, which was the method followed by all providers at 5 of the 6 clinics. At two clinics there were cases where providers first used that applicator to transfer the varnish to their glove, which removed the need to keep reaching back to the packet to get more on the applicator. Our analysis found this method to be faster. However, this method may not work as well for older children with more teeth since a higher percentage of the varnish provided in the packet is needed to cover those teeth. At one of the two clinics, the provider using that method only did so on her younger patients, while at the other clinic all applications were done with that method. One provider at clinic F used her gloved finger to apply the FV to the children’s teeth. Finally, our analysis found that head position did not have a statistically significant impact on the Standard Time. This may be because most providers have already determined the best head position to use for children of various ages.

For patient age, the youngest children tended to be the fastest, likely since they had the fewest teeth and were easiest to hold still. The slowest age group was the 3 years well visit. It was somewhat surprising that patient cooperation did not impact the time in a statistically significant way. However, when the younger children are very resistant, it is sometimes easier to apply the varnish since they tend to open their mouths wide when crying. In comparison, older children need to be coerced to open their mouths when they are upset, so a resistant older child may take longer. This relationship may be more apparent in a larger data set.

Delays also impacted the Standard Time. Clinics experienced, on average, 1.45 delays (i.e., foreign elements) per patient. The three most common delays of needing to reposition the child, child resistance, and additional conversations with parents accounted for 52% of all delays. The other most common delays were needing to pry the child’s mouth open, needing to get the child’s attention, problems with the paper on the exam table, and a general lack of urgency from the provider. Having to struggle with these delays leaves less time to fit other protocols in during a well visit, but these delays are accounted for in the times reported here.

## 7| Social Implications

Our FV study among six primary care clinics in Georgia found that the median Standard Times varied by clinic, ranging from 1 minute and 8 seconds to 2 minutes and 47 seconds. Clinics with less FV treatment experience took significantly longer to administer FV than more experienced clinics, but the overall mean and median were approximately two minutes. The VA ratio of actual FV application time accounts for about 30% of the total FV process time. The top reasons for delays include repositioning the child, a child’s refusal or resistance, and extra conversation with parents. Pediatric well visits have many tasks that must, or could be, completed within the limited time of the visit. Deciding which to include by balancing the health impact and time required for each procedure is essential to optimizing health. FV application has already been shown to be highly effective. This research further supports the inclusion of FV treatments as a common practice in primary care clinics by reducing the concern of the time that the procedure will take.

## Figures and Tables

**Table 1.** Patient characteristics by clinic.

			Percent of children in each well-visit categoriy							Percent of children by cooperation level		
Clinic	#Patients	%Male	<12mos	12mos	15mos	18mos	2year	3yr	4yr+	Easy	Moderate	Difficult
A	14	21%	7%	21%	14%	21%	14%	0%	21%	29%	43%	29%
B	20	60%	0%	60%	0%	10%	30%	0%	0%	25%	30%	45%
C	20	75%	15%	20%	15%	5%	10%	15%	20%	20%	30%	50%
D	14	36%	14%	14%	14%	0%	14%	7%	36%	57%	29%	14%
E	19	32%	11%	16%	5%	11%	21%	26%	11%	42%	26%	32%
F	7	57%	14%	29%	14%	14%	0%	29%	0%	14%	71%	14%
Total	94	48%	10%	28%	10%	10%	17%	12%	15%	32%	34%	34%

**Table 2.** FV application methods by clinic.

					Assigned allowance for delay				FV method			Patient positioning			
Clinic	Location	Practice size	FV Application Experience	#Patients	Low (5%)	Somewhat low (10%)	Somewhat high (15%)	High (20%)	FV dab on wrist	FV packet to Applicator	FV mixed to index finger	Reclined, Head in provders’s lap	Held by caregiver, upright position	Sitting upright un assistated	Reclined on exam table
A	Suburban	Small	Established	14	0%	64%	29%	7%	100%	0%	0%	29%	14%	14%	43%
B	Urban	Large	Established	20	25%	30%	45%	0%	10%	90%	0%	70%	10%	0%	20%
C	Urban	Small	Established	20	20%	60%	20%	0%	0%	100%	0%	0%	10%	45%	45%
D	Rural	Medium	Restarted	14	0%	57%	43%	0%	0%	50%	50%	0%	14%	36%	50%
E	Suburban	Medium	New	19	0%	74%	26%	0%	0%	100%	0%	42%	16%	16%	26%
F	Rural	Small	Less Consistent	7	0%	14%	43%	43%	0%	100%	0%	71%	14%	14%	0%
Total				94	10%	53%	33%	4%	17%	76%	7%	33%	13%	21%	33%

**Table 3.** FV time by clinic

		Standard Time (sec)				Application Time (sec)			Value-added (VA) ratio	
Clinic	#Patients	Median	Mean	St.Dev.	95% Conf. Int.	Median	Mean	StDev	Median	Mean
A	14	67.7	70.4	16.0	(61.2,79.7)	23.2	21.7	8.0	34.2%	30.9%
B	20	91.5	102.1	39.1	(83.8,120.4)	28.1	32.8	14.8	30.7%	32.2%
C	20	105.1	120.9	47.0	(98.9,142.9)	44.1	56.0	37.5	42.0%	46.3%
D	14	162.4	189.6	94.1	(135.3,244.0)	19.4	52.9	57.5	11.9%	27.9%
E	19	166.9	163.2	66.4	(131.2,195.3)	57.9	65.2	37.6	34.7%	39.9%
F	7	143.8	147.2	41.1	(109.2,185.2)	40.2	44.6	23.5	27.9%	30.3%
Total	94	**109.7**	**130.1**	**67.1**	**(116.4,143.9)**	**32.4**	**46.5**	**36.6**	29.6%	35.7%
